# High national rates of high-dose dopamine agonist prescribing for restless legs syndrome

**DOI:** 10.1093/sleep/zsab212

**Published:** 2021-08-21

**Authors:** John W Winkelman

**Affiliations:** Massachusetts General Hospital, Boston, MA, USA

**Keywords:** dopamine agonists, restless legs syndrome, pramipexole, rotigotine, ropinirole, augmentation

## Abstract

**Study Objectives:**

Long-term dopamine agonist (DA) use in restless legs syndrome (RLS) is associated with augmentation, a dose-related symptom worsening leading to further dose escalation to manage RLS. This study investigated rates and factors of high-dose DA prescribing in US RLS patients.

**Methods:**

This retrospective analysis examined data from a US longitudinal prescriptions database (October 2017–September 2018). Patients diagnosed with RLS (ICD-10 G255.81) without Parkinson’s disease who were prescribed ropinirole, pramipexole, and/or rotigotine were included. Daily DA dosage was categorized: LOW/MID (US Food and Drug Administration [FDA]-approved/guideline or slightly above FDA-approved [pramipexole]); HIGH (101%–149%); VERY HIGH (>150%). Patient counts were converted to US national estimates. Logistic regression of patient counts evaluated factors associated with HIGH/VERY HIGH DA dosing.

**Results:**

Of 670,404 RLS patients (131,289,331 therapy days), 58.8% were prescribed DA therapy. Overall, 19.1% of RLS patients were prescribed DAs above maximum FDA-approved/guideline daily doses—over half of these were >150% maximum recommended doses; 67.6% of HIGH/VERY HIGH-dose prescriptions were pramipexole (OR [95% CI] pramipexole vs ropinirole, 5.8 [5.7 to 6.0]). The highest 1% of DA prescriptions were ≥10× the FDA-recommended maximum daily dose. Rates of HIGH/VERY HIGH DA dosing increased with patient age. Twice as many neurologists (31.1%) prescribed HIGH/VERY HIGH doses vs other specialties (OR [95% CI], 2.1 [1.2 to 2.0]).

**Conclusions:**

Approximately 20% of DA-treated RLS patients were prescribed doses above the approved and guideline daily maximum. Pramipexole, Neurology as specialty, and patient age were independently associated with HIGH/VERY HIGH DA dosing. Increased education is warranted regarding risks of high-dose DA exposure in RLS.

**Statement of Significance**

Use of long-term dopamine agonists (DAs) in restless legs syndrome (RLS) is commonly associated with augmentation, a dose-related iatrogenic worsening of RLS, which can lead to further DA dose escalation to manage worsening symptoms. This article reports the results from an observational study that investigated the rates and factors associated with prescription of high-dose DA in patients with RLS in the US using data from a longitudinal US national prescription database. Overall, 19.1% of patients with RLS were prescribed DA doses above the maximum US Food and Drug Administration-approved/guideline daily doses; 10.5% of all DA prescriptions were >150% of the maximum recommended dose. These findings suggest that enhanced prescriber education on acute and long-term risks of high-dose DA exposure for RLS patients is warranted.

## Introduction

Restless legs syndrome (RLS) affects approximately 2.5% of the general population (approximately twofold greater incidence in women than men) [1–3], and is characterized by dysesthesias and irresistible urge to move the legs. Symptoms develop during inactivity, particularly at night, and interfere with sleep [1, 4]. RLS affects quality of life and is associated with increased all-cause mortality [2, 5–8].

Dopaminergic agents (levodopa and dopamine agonists [DAs]) have been the mainstay of RLS treatment for three decades [1, 9, 10]. Robust efficacy and tolerability data exist for the three DAs currently approved by the US Food and Drug Administration (FDA) for RLS: immediate-release oral ropinirole [11–16] and pramipexole [17–22] and transdermal patch rotigotine [23–25]. Maximum daily DA doses approved for RLS are much lower than those for Parkinson’s disease [16, 22, 26]. Dose-related acute adverse events (AEs) associated with DA use in RLS include nausea, somnolence, impulse control disorders [1, 9], and other psychiatric AEs [27].

Long-term DA use in RLS is associated with loss of efficacy and augmentation [28, 29], a progressive symptom exacerbation, with earlier daytime appearance of symptoms, increased symptom severity, shorter duration of medication benefit, and/or symptom spread to upper extremities [28]. Cross-sectional primary care and specialty clinic population prevalence estimates of RLS augmentation with DAs are 20%–30% [30]. Higher DA doses are associated with greater risk and severity of augmentation [29, 31, 32]. A common clinical decision to address the loss of efficacy or earlier onset of RLS symptoms is to increase the dose, which often produces a temporary improvement in RLS symptoms. However, as higher DA doses are associated with greater risk and severity of augmentation, RLS symptoms often increase thereafter, leading to a cycle of progressively worsening augmentation and, ultimately, prescription of DAs at doses far higher than are approved for RLS.

To better understand DA dosing in RLS, we examined data from a longitudinal prescription database to determine how frequently DA prescriptions exceed the FDA-approved and guideline limits, and factors associated with high and very high dosing.

## Methods

### Standard protocol approvals, registrations, and patient consents

This was a retrospective analysis of a longitudinal prescription database; therefore, the following information relating to patient consent was not applicable to this study: approval by an ethical standards committee, identification of licensing committee approving the study, acquisition of written informed consent from individual prescription record holders, or consent to disclose participants’ identifiable information.

### Data source

LRx is a longitudinal prescription database subset of the National Prescription Audit database and captures approximately 150 million unique deidentified patients from >1 million prescribers. LRx covers approximately 65% of all retail and mail-order prescriptions in the United States. Data can be projected to the US population to provide national estimates of patient exposure to a particular drug or drug treatment group.

### Patients, reporting period, treatments

Patients in the United States treated with marketed products for RLS between October 2017 and September 2018 were identified from LRx. Patients were included if they had a diagnosis of RLS (ICD-10 25.81) and excluded if they also had a diagnosis of Parkinson’s disease (ICD-9 332, 332.0, 332.1; ICD-10 G20, G21, G21.1, G21.11, G21.19, G21.2, G21.3, G21.4, G21.8, G21.9). Prescriptions for any RLS product (DAs: pramipexole, ropinirole, rotigotine; non-DAs: gabapentin enacarbil and off-label treatments [amantadine, cabergoline, carbamazepine, clonazepam, codeine sulfate, gabapentin, pregabalin, tramadol]) were tracked and reported along with fill date, product strength, days’ supply, and quantity dispensed. The treatment assigned to each patient was based on the longest treatment episode (i.e. the longest number of consecutive days on the exact same treatment/treatment combination and dose level).

### Dopamine agonist dose-level categories

Dopamine agonist dose categories were based on FDA-approved and guideline-recommended DA dosing (Table 1): LOW/MID, which includes the range of FDA-approved/guideline maximum recommended doses; HIGH, 101% to 149% of FDA-approved/guideline maximum recommended doses; and VERY HIGH, ≥150% of FDA-approved/guideline maximum recommended doses. For pramipexole, the upper limit of the LOW/MID dose was set at 0.75 mg (above the FDA-approved dose of 0.5 mg) because most clinical guidelines recommend doses up to this level. When DAs were prescribed in combination, doses were summed across the individual agents using algorithms to calculate equivalent doses: equivalence of 4:1 was used for ropinirole and/or rotigotine when prescribed in combination with pramipexole, and equivalence of 1:1 when ropinirole and rotigotine were prescribed in combination.

**Table 1.** DA therapy dose-level grouping

DA dose (mg)	LOW/MID (<FDA MAXIMUM DOSE)	HIGH (101%–149% FDA maximum dose)	VERY HIGH (≥150% FDA maximum dose)
Pramipexole [22, 41]	0–≤0.75	>0.75–≤1.25	>1.25
Ropinirole [16]	0–≤4.0	>4–≤6.0	>6.0
Rotigotine [26]	0–≤3.0	>3.0–≤6.0	>6.0

DA, dopamine agonist.

### Analyses to determine factors associated with HIGH/VERY HIGH dopamine agonist dosing

To determine factors associated with HIGH/VERY HIGH DA dosing, data were examined by specific DA, age group (0–19 to >79 years by decade; <54 years and ≥54 years), prescriber (neurology, sleep medicine, primary care, psychiatry, nurse practitioner [NP]/physician assistant [PA], pain medicine, rheumatology, pediatrics, other; new prescriber vs same prescriber), treatment category (naive, continued, restart, new-to-brand), and DA dose level of first and last recorded prescription for each patient (dose progression).

### Treatment category assignment

For treatment category, the prescribing history of each patient in the 24 months prior to the first prescription in the reporting period (index prescription) was examined. If there was no prior DA prescription, the patient was assigned to the “naive” treatment category. If there was no prior prescription for the same DA as the index prescription, the patient was assigned to the “new-to-brand” category. If there was a prior prescription for the same DA and the run-out date (fill date + 1.5 ×days’ supply) overlapped with the index prescription, the patient was assigned to the “continued” treatment category. If there was a prior prescription for the same DA, but with a gap between the run-out date and the index prescription, the patient was assigned to the “restart” treatment category.

### Lorenz analyses

A Lorenz analysis is used to characterize population-level utilization of a specific drug by examining the share of total volume of that drug by the highest 1% or 10% of users with >120 days of therapy [33]. High values demonstrate a skewed utilization pattern and is characteristic of drugs of abuse, such as high-potency opioids or short-acting benzodiazepines. We have modified this analysis by assessing the percentage share of total DA dose usage by the highest 1% and 10% of DA users.

### Statistical analyses

Raw patient counts were converted to US national estimates using projection factors for each prescription based on the number of prescriptions in the National Prescription Audit database for a particular product. These were based on number of prescriptions in LRx and National Prescription Audit databases for a particular product within the month. Projection factors for each patient were based on the average of projection factors for their prescriptions within the study period.

Descriptive statistics were used to evaluate the proportion of patients prescribed RLS treatment (overall and by dose level). Logistic regression (odds ratio [OR], 95% CI) was performed on raw patient counts to evaluate factors associated with HIGH/VERY HIGH DA dosing. Interaction analyses were performed on potential predictors of HIGH/VERY HIGH DA dosing identified through logistic regression.

### Data availability statement

All data collected for this study may be shared with any qualified investigator at the discretion of the author. All data requests should be made directly to the author for consideration.

## Results

### Sample size and patient demographics

Within the 12-month reporting period (October 2017 to September 2018), 485,565 patients with RLS, constituting 95,617,127 treatment days, were identified from LRx. This projected to a US national estimate of 670,404 patients and 131,289,331 therapy days. Approximately two-thirds of patients were female. Mean age overall was 62.2 ± 14.7 years; one-third of patients were aged >69 years and <8% of patients were aged <40 years (Table 2).

**Table 2.** RLS prescription population demographics, projected numbers from 485,565 individuals

	n (%)	Mean (SD)
Sex		
Female	460,397 (68.7)	
Male	210,007 (31.3)	
Age (years)		
All	670,404	62.2 (14.7)
0–19	4184 (0.6)	12.2 (5.3)
20–29	12,343 (1.8)	25.6 (2.7)
30–39	35,643 (5.3)	35.3 (2.8)
40–49	77,594 (11.6)	45.1 (2.8)
50–59	138,034 (20.6)	54.9 (2.8)
60–69	174,807 (26.1)	64.5 (2.9)
70–79	146,330 (21.8)	75.0 (2.8)
>79	81,468 (12.2)	83.4 (1.9)

RLS, restless legs syndrome.

### Treatment overview

Roughly 60% (394,482/670,404) of patients with RLS were prescribed any DA therapy either alone or in combination, with or without concomitant non-DA therapy, and 41.2% (275,922/670,404) were prescribed only non-DA therapy (Table 3). Of all patients prescribed DA therapy for RLS, 70.7% (279,071/394,482) were prescribed DA therapy without concomitant non-DA therapy. Very few patients were taking multiple DAs (0.6% [2437/394,482]; Table 3). Ropinirole was the most commonly prescribed DA (61.5% of patients [242,428/394,482]), followed by pramipexole (37.1% [146,254/394,482]), and rotigotine (0.9% [3363/394,482]).

**Table 3.** Percentage of patients receiving prescriptions for RLS by dose level

	Patients, n/N (%)			
Treatment	Any dose level	LOW/MID (<FDA maximum)	HIGH (101%–149% FDA maximum)	VERY HIGH (≥150% FDA maximum)
Total, *n*	670,404			
Non-DA therapy only	275,922/670,404 (41.2)			
Any DA therapy	394,482/670,404 (58.8)	319,423/394,482 (81.0)	33,747/394,482 (8.6)	41,311/394,482 (10.5)
	**Any dose level**	**LOW/MID**	**HIGH**	**VERY HIGH**
DA therapy only	279,071/394,482 (70.7)	228,424/279,071 (81.9)	23,027/279,071 (8.3)	27,619/279,071 (9.9)
DA plus concomitant non-DA therapy	115,411/394,482 (29.3)	90,998/115,411 (78.8)	10,720/115,411 (9.3)	13,692/115,411 (11.9)
	**Any dose level**	**LOW/MID**	**HIGH**	**VERY HIGH**
DA* monotherapy	392,045/394,482 (99.4)	318,709/392,045 (81.3)	33,202/392,045 (8.5)	40,134/392,045 (10.2)
Pramipexole*	146,254/394,482 (37.1)	95,488/146,254 (65.3)	21,737/146,254 (14.9)	29,029/146,254 (19.8)
Ropinirole*	242,428/394,482 (61.5)	221,092/242,428 (91.2)	10,469/242,428 (4.3)	10,867/242,428 (4.5)
Rotigotine*	3363/394,482 (0.9)	2129/3363 (63.3)	996/3363 (29.6)	238/3363 (7.1)
DA combination therapy*^,^†	2437/394,482 (0.6)	713/2437 (29.3)	545/2437 (22.4)	1177/2437 (48.3)

*With or without concomitant non-DA therapy.

^†^When combination therapy was prescribed, doses were added across the individual agents using the pramipexole equivalent daily dose; pramipexole dose equivalence for ropinirole and rotigotine at 4:1, and ropinirole/rotigotine dose equivalence at 1:1.

DA, dopamine agonist; RLS, restless legs syndrome.

**Figure 1.**
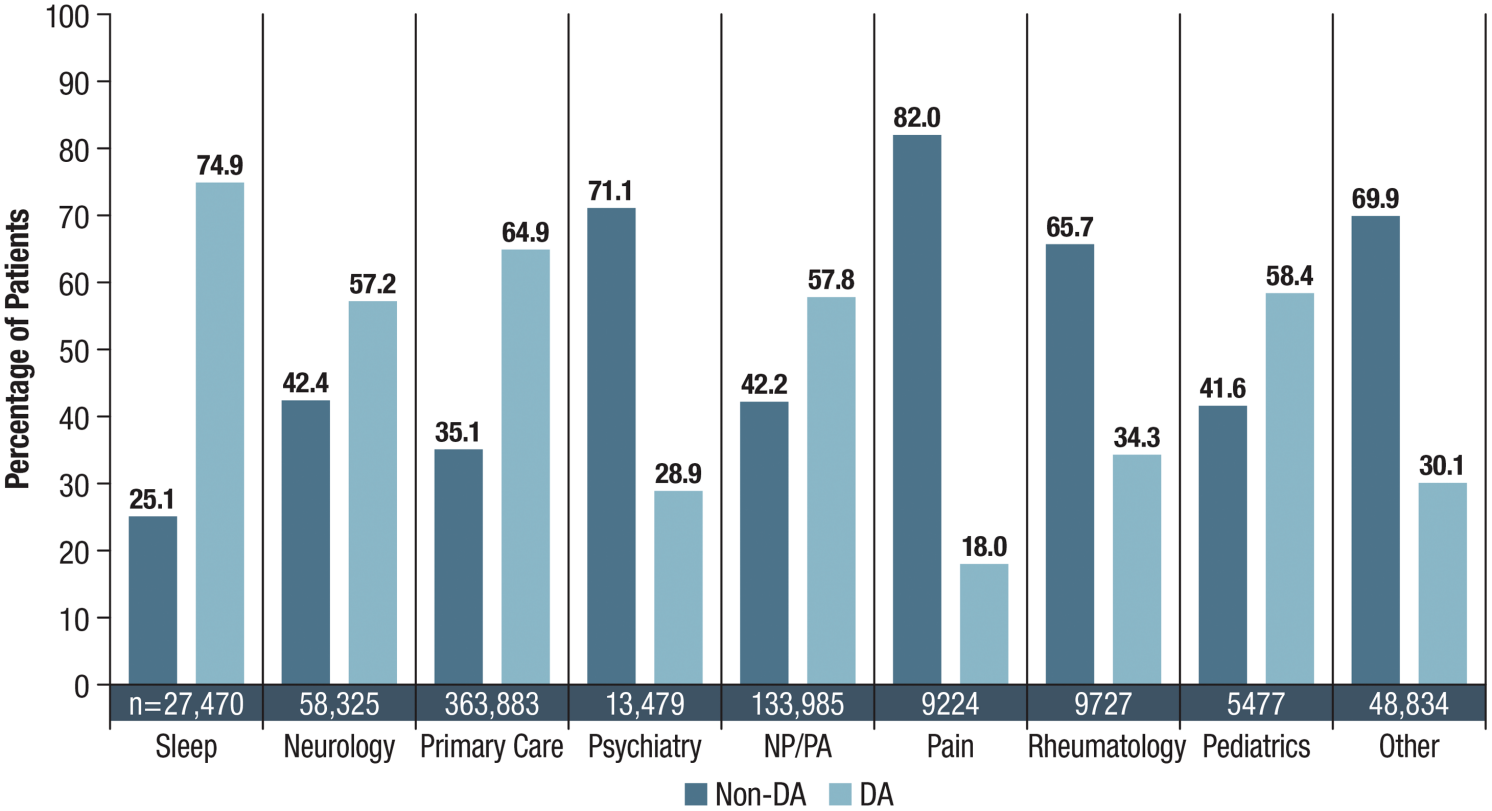
Patients (%) receiving Non-DA or DA therapy (± concomitant DA therapy) by prescribing specialty. DA, dopamine agonist; NP, nurse practitioner; PA, physician assistant.

Greater than 50% of patients (54.3%; 363,883/670,404) were prescribed RLS medication treatment by primary care physicians, followed by NPs/PAs (20%; 133,985/670,404), neurologists (8.7%; 58,325/670,404), and sleep specialists (4.1%; 27,470/670,404). The prescribing patterns for DA therapy with or without concomitant non-DA therapy vs non-DA therapy alone varied by specialty. Sleep specialists, primary care physicians, pediatricians, NP/PAs, and neurologists (in descending order) prescribed DA therapy more than non-DA therapy (Figure 1).

### HIGH (101%–149% maximum recommended)/VERY HIGH (≥150% FDA maximum recommended)-dose dopamine agonist therapy

For any DA therapy, with or without concomitant non-DA therapy, 19.0% of patients (75,058/394,482) were prescribed HIGH/VERY HIGH DA doses and 10.5% (41,311/394,482) were prescribed VERY HIGH DA doses (Table 3). The patient percentage prescribed HIGH/VERY HIGH DA doses was similar for DA therapy with and without concomitant non-DA therapy (21.2% [24,412/115,411] and 18.1% [50,646/279,071], respectively; Table 3).

Of all HIGH/VERY HIGH DA dose prescriptions, 67.6% ([21,737 + 29,029]/ [33,202 + 40,134 + 545 + 1177] = 50,766/75,058) were for pramipexole; 34.7% (50,766/146,254) of pramipexole prescriptions were above FDA-approved/guideline-recommended doses and nearly 20% (19.8%, 29,029/146,254) of pramipexole prescriptions were for VERY HIGH doses (>1.25 mg; Table 3). The OR (95% CI) for HIGH/VERY HIGH–dose pramipexole vs HIGH/VERY HIGH–dose ropinirole was 5.8 (5.7–6.0) (Supplementary Table e-1). Rotigotine and DA combination therapy were similarly prescribed at HIGH/VERY HIGH doses much more commonly than ropinirole (Table 3 and Supplementary Table e-1).

**Figure 2.**
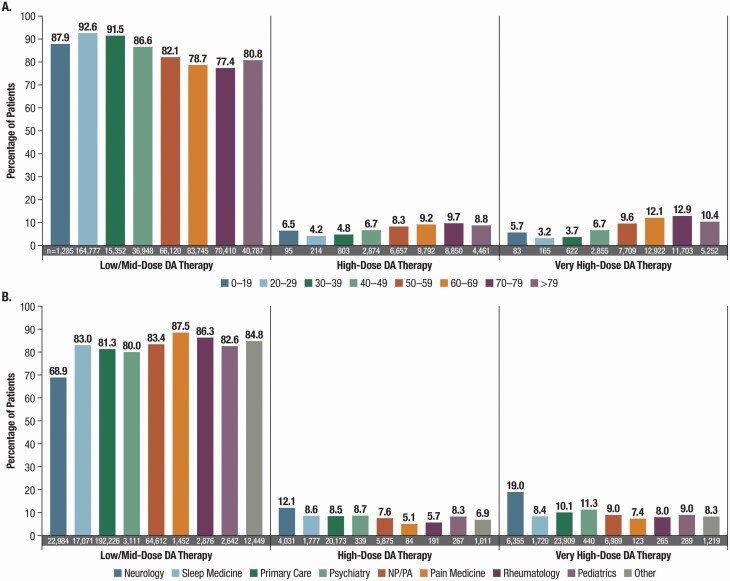
Patients (%) prescribed DA therapy by dose level and (A) age group (B) prescribing specialty. DA, dopamine agonist; NP, nurse practitioner; PA, physician assistant.

Male sex was weakly associated with HIGH/VERY HIGH–dose DA prescribing (OR, 1.2 [95% CI, 1.1 to 1.2]; Supplementary Table e-1). HIGH/VERY HIGH DA dosing increased with patient age, with highest rates occurring in the 70- to 79-year age group (9.7% prescribed HIGH- and 12.9% VERY HIGH–dose DAs; Figure 2A). The OR (95% CI) for patients aged ≥54 years vs <54 years was 1.8 (1.7 to 1.8) (Supplementary Table e-1).

Of all patients prescribed HIGH/VERY HIGH DA doses, 58.7% of prescriptions were by primary care physicians, 16.9% by NP/PAs, 13.8% by neurologists, and 10.8% by sleep specialists (Figure 2B). Neurologists prescribed HIGH/VERY HIGH DA doses to the greatest proportion of their patients: 31.1% (10,386/33,370), whereas sleep specialists prescribed such doses less frequently (17.0% [3497/20,568]) and pain specialists prescribed HIGH/VERY HIGH doses the least frequently (12.5% [207/1659]) among the specialties included in this study (Figure 2B). The OR (95% CI) of HIGH/VERY HIGH DA dose prescribing by neurologists was double that of other specialties (2.1 [1.2 to 2.0]; Supplementary Table e-1). The percentage of patients who were prescribed VERY HIGH DA doses by neurologists (19%, 6355/33,370) was almost double that of all other specialties (Figure 2B). Neurologists were nearly 10 times as likely to prescribe pramipexole at HIGH/VERY HIGH doses as all other specialists prescribing ropinirole (OR, 9.7 [95% CI, 9.2 to 10.2]; Supplementary Table e-1).

**Figure 3.**
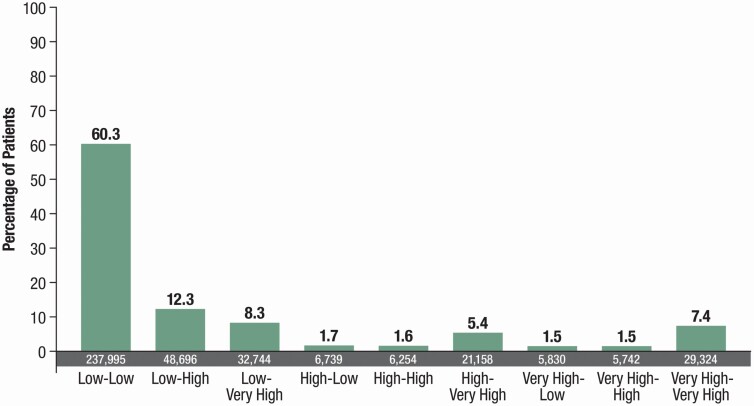
DA dose level progression of patients between first and last treatment episode. DA, dopamine agonist.

Approximately 25% of patients (24.4%, 54,908/225,430) on continued DA therapy (those with a prior recent prescription for the same DA) were prescribed HIGH/VERY HIGH DA doses, and patients on continued therapy were almost four times as likely to receive HIGH/VERY HIGH doses vs patients naive to DA therapy (OR, 3.7 [95% CI, 3.6 to 3.8]). Dose escalation was often rapid, with 12.3% of all patients receiving DAs for RLS progressing from LOW/MID dosing to HIGH, 8.3% from LOW/MID to VERY HIGH, and 5.4% from HIGH to VERY HIGH during the 12-month reporting period (Figure 3). Only 3.2% of patients had any reduction in DA dose level over the 12-month reporting period (Figure 3).

All prescriptions for DA monotherapy with or without concomitant non-DA therapy were converted to pramipexole equivalent doses and the composition of patients receiving the highest 1% of doses was examined (Supplementary Table e-2). In the highest 1%, mean ± SD dose was 5 ± 1.8 mg, median dose was 4.5 mg, minimum dose was 4 mg, and maximum dose was 60 mg. Approximately two-thirds of patients prescribed the highest 1% of DA doses were female and 88% were ≥54 years of age. Pramipexole prescriptions accounted for more than two-thirds of patients in the highest 1% of DA doses, with primary care as the prescribing specialty in 55% of patients. The mean dose of the highest 10% of pramipexole prescriptions was 2.0 mg. Most prescriptions were continuing care rather than new-to-brand.

The Lorenz analysis demonstrated a very skewed distribution of pramipexole dosing, with the highest 1% of pramipexole users constituting 10.4% of all combined dosing of that medication. The highest 10% of users of pramipexole constituted 45.0% of all prescribed supply.

## Discussion

Dopamine agonists have been first-line treatment for RLS for nearly 30 years owing to their nearly immediate efficacy and tolerability [1]. Unfortunately, over the long term, their efficacy often wanes and, even worse, they often produce augmentation, a dose-related, temporal, and anatomical extension of RLS symptoms [28]. Many clinicians’ response to such symptom exacerbation is to increase the dose of the DA, which may temporarily improve symptoms but worsens the underlying iatrogenic process, producing more severe augmentation of symptoms thereafter [28, 31]. Patients may end up with severe RLS symptoms much of the day, in both upper and lower extremities, unable to sleep for more than a few hours per day. The consequences of high-dose DA prescribing are thus not only an increased risk of acute AEs associated with these agents (orthostatic hypotension, peripheral edema, sleep attacks, impulse control disorders, hallucinations, and psychosis), but also a vicious cycle of progressive augmentation of RLS symptoms.

This is the first study that examines dosing of DAs on a large scale, population basis. In our sample of >500,000 RLS patients in the United States, nearly 20% were prescribed DA therapy at higher doses than those approved by the FDA or recommended by guidelines, and almost 10% of all RLS patients were prescribed DA therapy at VERY HIGH doses (>150% of FDA-approved/guideline-recommended doses). In particular, patients receiving pramipexole were almost 6 times as likely to be prescribed HIGH/VERY HIGH doses as those receiving ropinirole, with 20% receiving pramipexole at >150% above FDA-approved/guideline-recommended doses (equivalent to a ropinirole dose of >5 mg). This is even more meaningful given that we defined the low-dose pramipexole range to include up to doses of 0.75 mg, which is 50% higher than FDA-approved levels for RLS. Although rotigotine was prescribed much less commonly, when prescribed, patients were also 6 times more likely to receive doses higher than the FDA-approved/guideline recommendations when compared with those prescribed ropinirole. Why prescribing patterns for pramipexole and rotigotine were different from ropinirole is unclear. There is no evidence to suggest that augmentation rates or loss of efficacy are higher with pramipexole or rotigotine compared with ropinirole.

Interestingly, for all DA agents, ORs for HIGH/VERY HIGH prescribing were slightly higher in patients receiving DAs combined with non-DA therapy, suggesting that prescribers add on non-DA therapy when they have exhausted dose-level increases. Similarly, although it was infrequent that multiple DAs were prescribed simultaneously, in those cases, the risk of HIGH/VERY HIGH dosing was nearly 32 times that of ropinirole alone. This suggests that prescribers use different DA agents when they exhaust DA increases for a single agent, and that they are unaware of cumulative augmentation and AE risk when prescribing DAs in combination. Alternatives to DAs, calcium channel α _2_δ ligands (gabapentin enacarbil, pregabalin, and gabapentin) are also often prescribed for RLS based on demonstrated efficacy in clinical trials, although only gabapentin enacarbil is currently approved by the FDA [31]. However, side effects of these medications (sedation, dizziness, weight gain) may limit the doses of these medications. Other therapeutic agents for RLS include opioids [34, 35], though side effects and concerns about misuse/abuse limit the value of these medications, and supplemental iron (delivered orally or intravenously), which has been shown to be efficacious in RLS patients with low or low-normal serum ferritin levels [9, 36].

HIGH/VERY HIGH DA prescribing increased with patient age. In accordance with the known demographics of RLS, the overwhelming majority of patients were >40 years of age, with more than one-third >69 years of age [2]. Patients ≥54 years of age were almost twice as likely to be prescribed DAs at HIGH or VERY HIGH doses as patients <54 years of age. Association of patient age with high DA dose prescribing may be related to either greater symptom severity or longer duration of treatment leading to increased incidence of augmentation [28, 29, 31, 37]. We also found that prescribing DA therapy vs non-DA therapy increases with patient age. Increased HIGH/VERY HIGH DA dose prescribing with increased patient age may be related to a willingness to prescribe DAs at these doses, given concerns about the side effects of calcium channel α _2_δ ligands in older patients, including altered mental status and increased incidence of falls [31, 38].

Neurologists were nearly twice as likely as all other specialists to prescribe DAs at levels higher than FDA-approved/guideline-recommended doses, with 12.1% prescribing DAs at HIGH doses and nearly 20% prescribing at VERY HIGH doses. This could be related to the idea that neurologists treat patients with more severe symptoms, therefore requiring higher DA doses. However, if this were the case, one would expect such high dose-level prescriptions to be equally common for sleep medicine specialists, which was not observed. Another explanation could be that neurologists are accustomed to prescribing much higher doses of DA therapy for patients with Parkinson’s disease, therefore leading to increased comfort with higher-dose DAs in patients with RLS. However, although such doses are approved for Parkinson’s disease, they were never tested in patients with RLS. More importantly, although the acute side effects of high DA doses in Parkinson’s disease and RLS may be similar and problematic, augmentation is a uniquely harmful iatrogenic issue in patients with RLS.

Primary care physician prescribers accounted for almost 50% of all medications prescribed for RLS. They also prescribed the highest numbers of HIGH/VERY HIGH dose DA prescriptions: 58.7% of patients prescribed HIGH/VERY HIGH DA doses were prescribed by primary care. Specialties that prescribed DAs over non-DA therapy were also the specialties that prescribed a higher percentage of HIGH/VERY dose DA therapy proportionally, namely primary care, NP/PAs, neurology, and sleep medicine. This suggests that comfort/familiarity with DA therapy in RLS leads to less caution with DA dose increases.

Continued prescriptions were almost four times as likely to be at HIGH/VERY HIGH doses (24.4%) compared with prescriptions in patients who were naive to DA therapy or switching to a new DA therapy. This suggests that patients are not initiated on HIGH/VERY HIGH doses and that DA dosing escalates with continued prescribing of the same agent, most likely because of loss of efficacy or as a consequence of augmentation. Although the time course of loss of efficacy and augmentation with DAs in RLS is thought to occur over a matter of years, dose progression in our utilization data suggests that this may not be true; 25% of all patients progressed to a higher dose-level category from first to last prescription over the 1-year reporting period. However, it should be noted that this database captured information for patients who were at varying stages of their disease course. Furthermore, 7.4% of patients remained at VERY HIGH dose levels throughout the study period and very few patients experienced a reduction in prescribed DA dose.

Lorenz analysis is commonly used to characterize population-level drug utilization and has been used to demonstrate heavy use of prescribed drugs of abuse [33, 39]. Our modified Lorenz analysis demonstrates a highly skewed distribution of pramipexole doses in those with RLS, with the highest 1% and 10% of all prescriptions’ doses accounting for 10.4% and 45.0% of all supply of that medication. These values are well below those for high-potency, short-acting opioids but similar to those of alprazolam and zolpidem [39].

It is possible that all categories of RLS medications, not just DAs, are frequently dosed above recommended maximum doses, leading to excess side effects for RLS patients. However, data from a large observational study of opioids contradicts this, as the median dose in that cohort was 30 morphine milligram equivalents (MME) [40]. Those with MME > 50 were most likely to have comorbid pain conditions. Information on the dosing of alpha-2-deltas is not available, but insurance reimbursement limitations maintain dosing of gabapentin enacarbil at its FDA-approved level for RLS.

There are limitations to this study that are inherent to any retrospective, observational study (endpoints not prespecified; heterogeneous patient population). Detailed patient history on disease duration, symptom severity, treatment duration, treatment pathway, and reasons for change in therapy or dose level are unknown; therefore, any conclusion is tentative. Lastly, it is not known how many patients were taking levodopa/carbidopa because these data were not collected; however, it should be noted that a PD diagnosis was a criterion for exclusion from this study.

In conclusion, it is clear from these data that different specialties prescribe medications for RLS therapy differently and, when prescribed, DA dose is often escalated to a range that is higher—and often much higher—than that recommended by the FDA. Data suggest that prescribers, when faced with a patient with worsening symptoms, increase the dose of DA therapy, which can lead to or exacerbate augmentation, akin to putting out a fire with gasoline. This prescribing pattern highlights the need for prescriber education about the risks of high-dose DA use and a need for increased awareness of the occurrence and subsequent management of augmentation in patients with RLS.

## Supplementary Material

zsab212_suppl_Supplementary_Materials
