# Supplementary material for: High national rates of high-dose dopamine agonist prescribing for restless legs syndrome
Source: Sleep. 2021 Aug 21;45(2):zsab212. doi: 10.1093/sleep/zsab212 (PMC8842153; doi:10.1093/sleep/zsab212)
Supplement: zsab212_suppl_Supplementary_Materials [file zsab212_suppl_supplementary_materials.docx]

**Supplemental Materials**

***High National Rates of High-Dose Dopamine Agonist Prescribing for RLS***

John W. Winkelman

Massachusetts General Hospital, Boston, MA, USA

**Corresponding Author**

John W. Winkelman, MD, PhD

Chief, Sleep Disorders Clinical Research Program

Massachusetts General Hospital

One Bowdoin Square, 9th Floor

Boston, MA 02114-2790

Phone: 617-643-9101

Email: JWWINKELMAN@MGH.HARVARD.EDU

**Table e-1. Logistic Regression and Interaction Analyses for Potential Predictors of HIGH/VERY HIGH DA Dosing**

| **Odds Ratio Estimates** | | |
| --- | --- | --- |
| **Effect** | **Point Estimate** | **95% CI** |
| Sex |  |  |
| Male vs female | 1.2 | 1.1–1.2 |
| Prescribing specialty |  |  |
| Neuro vs other specialty | 2.1 | 1.2–2.0 |
| Age |  |  |
| ≥54 y vs <54 y | 1.8 | 1.7–1.8 |
| Treatment vs ropin only |  |  |
| DA combo + other non-DA | 32.7 | 27.6–38.7 |
| DA combo only | 25.6 | 22.5–29.1 |
| Pram + other non-DA | 7.2 | 7.0–7.4 |
| Pram only | 5.8 | 5.7–6.0 |
| Rotig + other non-DA | 7.3 | 6.4–8.3 |
| Rotig only | 6.3 | 5.7–7.1 |
| Ropin + other non-DA | 1.4 | 1.4–1.5 |
| Treatment category |  |  |
| Continued vs naive | 3.7 | 3.6–3.8 |
| Continued vs new-to-brand | 3.7 | 3.6–3.8 |
| Continued vs restart | 1.2 | 1.2–1.3 |
| Logistic regression with interaction |  |  |
| Specialty-age |  |  |
| Neuro/<54 y vs other/≥54 y | 1.1 | 1.0–1.2 |
| Neuro/≥54 y vs other/≥54 y | 2.1 | 2.0–2.2 |
| Other/<54 y vs other/≥54 y | 0.6 | 0.6–0.6 |
| Treatment-specialty |  |  |
| Pram only/neuro vs ropin only/other | 9.7 | 9.2–10.2 |
| Pram only/other vs ropin only/other | 6.0 | 5.9–6.2 |
| Pram + non-DA/neuro vs ropin only/other | 12.6 | 11.7–13.6 |
| Pram + non-DA/other vs ropin only/other | 7.4 | 7.2–7.7 |
| Ropin only/neuro vs ropin only/other | 2.5 | 2.4–2.7 |
| Ropin + non-DA/neuro vs ropin only/other | 3.0 | 2.8–3.2 |
| Ropin + non-DA/ other vs ropin only/other | 1.4 | 1.4–1.5 |
| Rotig only/neuro vs ropin only/other | 9.3 | 7.9–10.8 |
| Rotig only/other vs ropin only/other | 5.4 | 4.6–6.2 |
| Rotig + other non-DA/neuro vs ropin only/other | 10.6 | 8.5–13.2 |
| Rotig + other non-DA/other vs ropin only/other | 6.8 | 5.8–8.0 |

DA, dopamine agonist; pram, pramipexole; ropin, ropinirole; rotig, rotigotine.

**Table e-2. Highest 1% of DA Prescriptions**

| **99th Percentile** | **n (%)** | **Pramipexole Equivalent Dose, mg** | | | |
| --- | --- | --- | --- | --- | --- |
|  |  | **Mean ± SD** | **Median** | **Minimum** | **Maximum** |
| All | 3654 (100) | 5±1.8 | 4.5 | 4 | 60 |
| DA agent | | | | | |
| Pram only | 1615 (44.2) | 5±2 | 4.5 | 4 | 60 |
| Pram + non-DA | 865 (23.7) | 5±1.58 | 4.5 | 4 | 18 |
| Ropin only | 707 (19.3) | 5±1.7 | 4.5 | 4 | 24 |
| Ropin + non-DA | 455 (12.5) | 5±1.7 | 4.5 | 4 | 16 |
| Rotig only | 6 (0.16) | 4 | 4 | 4 | 4 |
| Rotig + non-DA | 6 (0.16) | 4±0.1 | 4 | 4 | 4 |
| Age, y | | | | | |
| <54 | 439 (12) | 5±3 | 4.5 | 4 | 60 |
| ≥54 | 3215 (87.9) | 5±1.6 | 4.5 | 4 | 24 |
| Sex | | | | | |
| Female | 2326 (63.7) | 5±1.9 | 4.5 | 4 | 60 |
| Male | 1328 (36.3) | 5±1.7 | 4.5 | 4 | 24 |
| Neurology vs other | | | | | |
| Neurology | 738 (20) | 5±1.8 | 4.5 | 4 | 18 |
| Other | 2916 (79.8) | 5±1.7 | 4.5 | 4 | 60 |
| By all specialties | | | | | |
| Primary care | 2009 (55) | 5±1.9 | 4.5 | 4 | 60 |
| Neurology | 738 (20) | 5.1±1.7 | 4.5 | 4 | 18 |
| NP/PA | 580 (15.9) | 5.1±1.7 | 4.5 | 4 | 16.5 |
| Sleep medicine | 130 (3.6) | 4.9±1.2 | 4.5 | 4 | 9 |
| Psychiatry | 36 (1) | 5.4±2.6 | 4.5 | 4 | 18 |
| Pain | 11 (0.3) | 5.3±2 | 4.5 | 4 | 10 |
| Rheumatology | 26 (0.6) | 5.1±1.7 | 4.5 | 4 | 10 |
| Pediatrics | 22 (0.6) | 4.7±0.9 | 4.5 | 4 | 6 |
| Other | 102 (3) | 4.9±1.2 | 4.5 | 4 | 10 |
| Change specialty | | | | | |
| No | 3342 (91.4) | 5±1.6 | 4.5 | 4 | 60 |
| Yes | 312 (8.5) | 5±1.6 | 4.5 | 4 | 16 |

DA, dopamine agonist; NP, nurse practitioner; PA, physician assistant; pram, pramipexole; ropin, ropinirole; rotig, rotigotine.
